# Nanogrooves for 2D and 3D Microenvironments of SH-SY5Y Cultures in Brain-on-Chip Technology

**DOI:** 10.3389/fnins.2020.00666

**Published:** 2020-06-24

**Authors:** Alex Bastiaens, Rahman Sabahi-Kaviani, Regina Luttge

**Affiliations:** Neuro-Nanoscale Engineering Group, Mechanical Engineering and the Institute for Complex Molecular Systems, Eindhoven University of Technology, Eindhoven, Netherlands

**Keywords:** SH-SY5Y cells, nanogrooves, neuronal differentiation, brain-on-chip, neurite length, microtunnel structures

## Abstract

Brain-on-chip (BOC) technology such as nanogrooves and microtunnel structures can advance *in vitro* neuronal models by providing a platform with better means to maintain, manipulate and analyze neuronal cell cultures. Specifically, nanogrooves have been shown to influence neuronal differentiation, notably the neurite length and neurite direction. Here, we have drawn new results from our experiments using both 2D and 3D neuronal cell culture implementing both flat and nanogrooved substrates. These are used to show a comparison between the number of cells and neurite length as a first indicator for valuable insights into baseline values and expectations that can be generated from these experiments toward design optimization and predictive value of the technology in our BOC toolbox. Also, as a new step toward neuronal cell models with multiple compartmentalized neuronal cell type regions, we fabricated microtunnel devices bonded to both flat and nanogrooved substrates to assess their compatibility with neuronal cell culture. Our results show that with the current experimental protocols using SH-SY5Y cells, we can expect 200 – 400 cells with a total neurite length of approximately 4,000–5,000 μm per 1 mm^2^ within our BOC devices, with a lower total neurite length for 3D neuronal cell cultures on flat substrates only. There is a statistically significant difference in total neurite length between 2D cell culture on nanogrooved substrates versus 3D cell culture on flat substrates. As extension of our current BOC toolbox for which these indicative parameters would be used, the microtunnel devices show that culture of SH-SY5Y was feasible, though a limited number of neurites extended into microtunnels away from the cell bodies, regardless of using nanogrooved or flat substrates. This shows that the novel combination of microtunnel devices with nanogrooves can be implemented toward neuronal cell cultures, with future improvements to be performed to ensure neurites extend beyond the confines of the wells between the microtunnels. Overall, these results will aid toward creating more robust BOC platforms with improved predictive value. In turn, this can be used to better understand the brain and brain diseases.

## Introduction

To study neuronal function and understand its role in brain physiology and pathology, *in vitro* neuronal models are advancing beyond the scope of conventional titer plate and Petri dish cultures to enhance the insights taken from such experiments. In particular, micro- and nanotechnology have allowed for the implementation of well-defined and small-scale platforms that provide neuronal cell cultures with both a more realistic *in vivo*-like microenvironment and the means to maintain, manipulate and analyze cells at a more detailed level compared to conventional methods. This has given rise to organ-on-chip (OOC) platforms, here specifically brain-on-chip (BOC) platforms. Considering the immense complexity of brain function, as well as the lack of therapeutic agents for many neurodegenerative diseases, BOC technology is showing potential to provide beneficial answers in studying brain function at the cellular and tissue scale for both a better fundamental understanding and toward the development of improved preclinical screening models.

In a BOC platform, the microenvironment can be engineered to exert physical control over how a neuronal cell culture is organized and analyzed. To study morphological aspects of neuronal differentiation, such as neuronal outgrowth (neurite) length and direction, compartmentalization and nanotopography are strategies that have shown promise as part of the BOC toolbox. Compartmentalization, as seen in microfluidic systems (Taylor et al., 2005; Park et al., 2009, 2014; Hosmane et al., 2010; Wheeler and Brewer, 2010; Jadhav et al., 2015) based on the principles of the Campenot chamber (Campenot, 1977), provides a geometric confinement of the neurites separated from the neuronal cell bodies. Nanotopography can influence cellular behavior (Hoffman-Kim et al., 2010; Limongi et al., 2013; Nguyen et al., 2016; Onesto et al., 2017) showing changes in neuronal differentiation and organization (Johansson et al., 2006; Bremus-Koebberling et al., 2012; Xie and Luttge, 2014) as these provide substrates with nanoscale features that replicate the nano-architecture of proteins in the extracellular matrix (ECM) (Kim et al., 2018).

In our previous work, we have investigated the effect of a range of nanogrooved patterns on the human neuroblastoma cell line SH-SY5Y. With our image-based screening method we found that certain pattern dimensions increase organization within the cell culture through the alignment of neurites on the underlying nanogrooved substrate, positively correlating to neuronal differentiation and outgrowth length (Bastiaens et al., 2018b, 2019a). Also, the effect of the nanogrooves is extended into the 3D microenvironment as seen for primary rat cortical cells and SH-SY5Y cells on a nanogrooved substrate and within a hydrogel, here growth factor reduced Matrigel (Bastiaens et al., 2019b). As a first step in the optimization procedure for the BOC system design and its robustness, the effect of implementing features such as nanogrooves was quantified for total neurite length in 2D and 3D neuronal cell culture on nanogrooved substrates as a baseline expectation.

In this work, we want to extend upon the findings for total neurite length as a baseline expectation from 2D and 3D neuronal cell cultures on flat substrates from previous work (Bastiaens et al., 2019b). This comparison has not yet been performed and aids the use of BOC prototypes in the neuroscience community. While nanotopography effects on 3D neuronal cell cultures have been investigated previously, these studies are typically limited to the direct interaction of cells with a 3D shape (Limongi et al., 2013), instead of a 3D scaffold microenvironment extending micrometers away from the substrate as seen in our work. In conjunction, we aim for a novel approach where the beneficial effects of nanogrooves on neuronal differentiation are combined with microfluidic compartmentalization.

The microfluidic chips were fabricated in PDMS by means of a soft lithography process. Chips consisted of microtunnels in two different configurations, with either parallel, linear channels or channels in a radial design with a joint intersection at the center. The parallel microtunnels were completed on either flat PDMS substrates or PDMS substrates with nanogrooves parallel to the microtunnels with the aim of raising the number and length of neurites within the microtunnels on nanogrooves. All microtunnels are connected to an inlet and outlet compartment. The inlet compartment serves as a reservoir for the neuronal cell culture. The inlet and outlet compartments are connected by these microtunnels and facilitating the direct placement of cells in the close vicinity of microtunnels by pipetting.

Our current results show that we can differentiate SH-SY5Y cells into the neuronal phenotype in our newly developed chip that employs both nanogrooves and microtunnels in case of the linear configuration, and keep these cells for longer cell culture times up to at least 21 days *in vitro* (DIV). For the geometrical confinement introduced here, neurites are shown to be able to grow into the microtunnels.

The overview for total neurite length as measured specifically for 2D and 3D neuronal cell cultures with nanogrooves, in combination with the first results shown for the chip with nanogrooves and microtunnels, add valuable knowledge toward implementing and optimizing the BOC toolbox. In turn, this will aid our endeavors to design BOC systems that can advance the understanding of brain function and neurodegenerative disease.

## Materials and Methods

### Nanogroove Fabrication

Nanogroove fabrication has been performed according to a previously published protocol by Xie and Luttge (2014). In brief, jet and flash imprint lithography (J-FIL) was used to pattern nanoresist on a standard double-sided polished silicon wafer with a 100 mm diameter and a bottom anti-reflective coating (DUV30J, Brewer Science, Rolla, MO, United States) layer applied using a quartz master. The quartz master was kindly provided by the Bijkerk group at the University of Twente. The nanoresist patterns consisted of nanogrooves ranging from a pattern periodicity of 200–2,000 nm with ridge widths of 100–1340 nm. The nanoresist patterns were used in thermal nanoimprint lithography to create a negative copy in cyclic olefin copolymer (COC; optical grade TOPAS 8007S-04, Topas Advanced Polymers, Frankfurt am Main, Germany). The COC template was used for repeated replication of the nanogrooves into a 100 μm layer of PDMS (Sylgard 184, Dow Corning, Midland, MI, United States) by means of spin coating PDMS onto the COC. PDMS was made at a ratio of 10:1 elastomer to curing agent. Specifically, for the results detailed in this work, nanogrooved PDMS substrates with a pattern period of 1,000 nm and a ridge width of 230 nm were used in neuronal cell culture as these patterns had shown the largest influence on neurite alignment (Bastiaens et al., 2019a). For 2D neuronal cell culture, the nanogrooved PDMS substrates including flat PDMS surface areas as control were placed in Petri dishes. Sterilization of the substrates was done by immersion in 70% ethanol. Subsequently, the devices were washed three times using sterile demineralized water prior to use in cell culture.

To create a 3D microenvironment on top of a nanogrooved substrate, fabrication was performed according to our previous work on nanogroove-enhanced 3D neuronal cell culture (Bastiaens et al., 2019b). The device allows for a microenvironment combining a hydrogel and the nanogrooved substrate, so that cells experience the *in vivo*-like cues from the hydrogel and the topographical cues of the nanogrooves. Here, we use growth factor reduced Matrigel as the hydrogel. In brief, nanogrooved PDMS substrates for these 3D neuronal cell cultures were obtained from the same COC template as described in the previous paragraph, and spin-coating the PDMS into a 100 μm layer. After removing the nanogrooved PDMS layer from the COC template, the nanogrooved pattern with 1,000 nm pattern period and 230 nm ridge width was punched from the PDMS substrates using a 10 mm diameter punch. A 10 mm and a 3 mm diameter punch were used to punch out a ring of flat PDMS from the flat areas surrounding the area of the nanogrooves on the PDMS substrate. Plasma oxidation using oxygen plasma at 20W for 30 s was performed using a plasma asher (EMITECH K1050X, Quorum, Laughton, United Kingdom) to bond the PDMS ring and PDMS nanogrooved substrate together directly after plasma oxidation. The combined PDMS layers were placed in an oven at 65°C for 1 h. The PDMS constructs on nanogrooves were then placed in a well plate for 3D neuronal cell culture. The PDMS constructs were prepared for cell culture by immersion in 70% ethanol and washed three times using sterile demineralized water.

The dimensions of the nanogrooved patterns used in the 2D and 3D culture devices described in this section were verified using atomic force microscopy (AFM; XE-100, Park Systems). A non-contact cantilever (PPP-NCHR, Park Systems) was used in tapping mode in conjunction with XEP software (Park Systems) to record the AFM data. Afterward, the profiles from the nanogrooved patterns were analyzed using Gwyddion software (Neèas and Klapetek, 2012).

### Microbioreactor Fabrication

For cell culture in hydrogel scaffolds with a diameter of 3 mm and up to 2 mm in height, a microfluidic assisted 3D microenvironment must provide sufficient nutrition and waste exchange. Such a microfluidic device was previously designed by us and coined a microbioreactor (Schurink and Luttge, 2013). In brief, the microbioreactor was designed to be able to place a cylindrical chamber for long-term 3D cell culture in a leakage-free manner on top of microelectrode arrays or other types of integrated readout systems. In case of optical readout using fluorescent microscopy the microbioreactor can also be sealed against a simple flat glass plate, which we did for this study. Here, a variant of the original design of the microbioreactors was applied, which has been also published by us previously (Bastiaens et al., 2018a). In more detail, microbioreactors were fabricated using soft lithography of PDMS in poly(dimethyl methacrylate) (PMMA) milled molds and assembly by a surface mount technique. The inclusion of a PES tube into the molded PDMS gaskets forms the culture chamber, which is similar in diameter as the wells of a standard 384-wells plate. However, here, the porous PES allows for microfluidic feedstock supply and waste removal. To form a PDMS sealing structure at the bottom opening of the microbioreactor construct (PDMS gasket and PES tube), first a thin PDMS layer was coated onto a PMMA plate with 3 mm diameter holes, the same size as the inner diameter of the PES tube. A doctor blade method was used by moving a scraper over the PMMA plate to remove the excess PDMS. Subsequently, the plate was placed onto a hotplate at 65°C for 17 min to partially cure the layer of PDMS. In a next step, microbioreactor constructs are positioned on the PDMS pre-coated PMMA plate with the hole aligned to the PES tube. The assembly was placed back onto the hotplate for another 2 h to cure fully. After curing the microbioreactor constructs are taken of the PMMA plate and the bottom surface was plasma oxidized as given in Section “Microbioreactor Fabrication” and immediately mounted to a 13 mm diameter microscope glass cover slip. The completed microbioreactors were prepared for culture experiments also by 70% ethanol sterilization and three washing steps as already described in Section “Microbioreactor Fabrication” above.

### Microtunnel Device Fabrication

Another 3D-type of cell culture microenvironment known in the literature is the so called microtunnel device. For this study, two different configurations were designed and fabricated. The first configuration consists of a linear arrangement of microtunnels running parallel to each other between an inlet and an outlet. The second configuration consists of a radial arrangement, with an inlet in the middle and microtunnels extending toward a set of outlets punched at a defined distance in the circumference of the inlet. The radial configuration would allow for compartmentalized neuronal cultures to connect to multiple other compartments with similar distribution and length of outgrowths in all directions.

The linear configuration allows for more straightforward quantification of neurite length and growth into the microtunnels as well as direct visual comparison between microtunnels connecting two compartments. A scale bar was added along the sides of the microtunnel area on the chip with ticks at a 500 μm interval for initial assessment of neurite growth during experiments. Several microtunnel dimensions were fabricated by photolithography with a height of 5 μm and a varying tunnel width of 10, 15, and 20 μm, respectively. The distance between the tunnels is either 50 or 100 μm.

The radial configuration was designed to maximize the opportunity for forming neurite connections from a single inlet to several distinguished compartments on the microchip.

**FIGURE 1:**
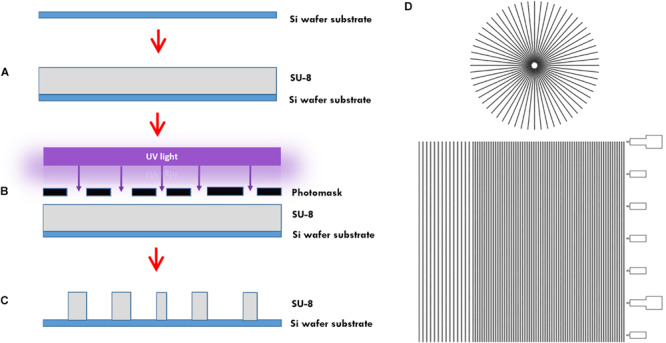
Microtunnel device fabrication using photolithography. **(A)** A SU-8 2050 negative photoresist (SU-8) was spin coated onto a silicon wafer. **(B)** A mask was placed onto pre-baked photoresist and the components were subjected to a collimated UV light source. **(C)** After a post-bake, the wafer is placed in developer to clear away the soluble, unexposed photoresist. This leaves a detailed design of the mask in the photoresist on the silicon wafer that can be used as a master for use in creating microtunnel devices using soft lithography with polydimethylsiloxane. **(D)** Part of the design of the mask, showing both the microtunnels in a linear and radial configuration. A scale bar was added along the linear microtunnels, with ticks at a 500 μm interval. Microtunnels were designed with a height of 5 μm and a tunnel width of either 10, 15, and 20 μm. The distance between the tunnels is either 50 or 100 μm.

In more detail, the microtunnels were fabricated using conventional photolithography (Figures 1A–C). A 5 μm layer of the negative photoresist SU-8 2010 (Microchem Inc., Newton, MA, United States) was spin-coated onto a 100 mm diameter silicon wafer (Figure 1A), pre-baked on a hotplate for 2 min at 95°C and exposed to 105 mJ cm^–2^ of collimated UV light source through a film photomask (designed in AutoCAD 2019, printed by CAD/Art Services Inc., Bandon, OR, United States) (Figures 1B,D), after which a post-exposure bake on a hotplate for 3 min at 95°C was performed and a bath of developer (mrDev-600, Microresist Technology GmbH) was applied. The microtunnel device master was subsequently hard baked on a hotplate for 5 min by 150°C (Figure 1C). Soft lithography was performed to transfer the microtunnels into PDMS using the same ratio of elastomer and curing agent as for all our other microdevices described in this paper. After pouring the PDMS onto the wafer with a height of approximately 5 mm it was cured at 65°C for 2 h.

Microtunnel devices were cut from the PDMS substrate and peeled-off from the master containing microtunnels of the various configurations. Inlet and outlets were punched using a 2 mm diameter biopsy tool. Subsequently, the microtunnel devices are either sealed with a flat layer of PDMS of 100 μm thickness or with the nanogrooved PDMS substrates, which were fabricated as described in Section “Microbioreactor Fabrication.” Device sealing is performed again by using oxygen plasma to activate the PDMS surfaces and direct bonding as described already in Section “Microbioreactor Fabrication.” For ease of handling, the bottom of the nanogrooved PDMS substrate and 35 mm diameter glass cover slips were also plasma oxidized with the same settings and subsequently bonded. The microtunnel devices with the radial configuration were plasma bonded directly to 35 mm diameter glass cover slips. These microtunnel microchips were prepared for cell culture also by ethanol and according to washing steps detailed already above.

To verify whether microtunnels would connect between inlets and outlet, and to verify the bonded PDMS components were free of leakage, a blue food dye was diluted in demineralized water at 20% (v/v) and was placed on the inlets of a linear and radial microtunnel device. The flow of dyed water through the microtunnels was observed through a microscope and a video was captured.

### SH-SY5Y Cell Culture

#### Coating of Devices

After sterilization and washing of the microdevices a fibronectin coating of 20 μg cm^–2^ fibronectin (Sigma-Aldrich, Zwijndrecht, Netherlands) in PBS (Westburg, Leusden, Netherlands) was applied to the culture surfaces of each device for half an hour. Upon aspirating of the coating solution, cells were immediately seeded.

#### 2D Cell Culture on Nanogrooves and Flat Substrates

The 2D cell cultures were performed as described in Bastiaens et al. (2018b). SH-SY5Y cells (94030304, Sigma-Aldrich) were thawed from liquid nitrogen and cultured in T75 flasks in standard medium, composed of DMEM/F12 medium (VWR) supplemented with 10% FBS (Bovogen, Keilor East, VIC, Australia) and 1% penicillin/streptomycin (Westburg) in an incubator at 37°C and 5% CO_2_. Upon reaching a confluency of 70-80% cells were detached using trypsin. The cells were seeded onto the nanogrooved and flat PDMS substrates at 1,500 cells cm^–2^ at 0 DIV and after 3 h of leaving the cells to adhere to the substrate, the standard medium was replaced with differentiation medium. The differentiation medium consisted of standard medium supplemented with 10 μM RA (Sigma-Aldrich) for 72 h to initiate differentiation and inhibit proliferation of the SH-SY5Y cells (Dwane et al., 2013; Teppola et al., 2016). The differentiation medium was changed once during that time. After 72 h, standard medium supplemented with 50 ng ml^–1^ BDNF (Sigma-Aldrich) was applied to the cells for 24 h to further enhance neuronal differentiation (Encinas et al., 2000; Teppola et al., 2016). From 4-21 DIV standard medium was used to maintain the cell culture, with medium refreshed every 2 days. At 21 DIV, cells were fixed by means of a 3.7% paraformaldehyde (Merck Millipore, Amsterdam, Netherlands) solution in PBS twice for 30 min. Five samples of nanogrooved and flat PDMS substrates were analyzed in the context of this work.

#### 3D Cell Culture on Nanogrooved Substrates

For the 3D nanogrooved PDMS devices, cells were handled similarly to the 2D experiments described above, which is also described in our previous work (Bastiaens et al., 2019b). Cells were, however, seeded on 0 DIV at 20,000 cells cm^–2^ and left to adhere for 3 h prior to adding a layer of growth factor reduced Matrigel (734-0269, VWR), which was thawed on ice, on top of the cell culture. The cultures were left in the incubator for 15 min for the hydrogel to gel, after which medium with 10 μM RA was added to the devices to start differentiation. This differentiation medium was refreshed once during that time. After 72 h, standard medium supplemented with 50 ng ml^–1^ BDNF was used instead of the medium supplemented with RA for 24 h. For 4–21 DIV standard medium was used to maintain the cell culture, with medium refreshed every 2 days. At 21 DIV, cells were fixed by means of a 3.7% paraformaldehyde solution in PBS for 1 h. Three samples of nanogroove-enhanced 3D cultures were analyzed in the context of this work.

#### 3D Cell Culture on Flat Substrates

For the 3D culture on flat substrates the microbioreactors (Bastiaens et al., 2018a) were utilized. In this case, cells were directly mixed with the growth factor reduced Matrigel at a concentration of 400,000 cells ml^–1^, with 15 μl of cell solution pipetted into each microbioreactor. Again, Matrigel was allowed to gel for 15 min in an incubator prior to adding medium and continuing the experiment up until fixing the samples at 21 DIV. Other than cell seeding density, the culture and fixing protocol for these devices was the same as for 3D cell cultures as described above. Three samples of 3D cultures on flat substrates were analyzed in the context of this work.

#### 3D Cell Culture in Microtunnel Devices

For 3D culture in microtunnels, SH-SY5Y cells are expected to extend their neurites into the microtunnels connected to the inlet. To ensure the likelihood for such phenomenon to occur cells are cultured by starting from a high cell seeding density of 100,000 cells cm^–2^. Other than cell seeding density, the culture and fixing protocol for these devices was the same as for 2D cell cultures as described above. Microtunnel devices were placed in six wells plates, each well containing five devices of either linear or radial configuration. During cell culture, the five microtunnel devices placed in the well were submerged in 2 ml of medium, to ensure sufficient nutrients could reach to each inlet containing cells.

### Immunofluorescence Staining and Imaging

Cells for the 2D and microtunnel devices were immunofluorescently stained using anti-β-Tubulin III (T8578, Sigma-Aldrich) and anti-mouse IgG Alexa Fluor 555 (A21424, Thermo Fisher Scientific) as primary and secondary antibody, respectively, to stain for neuronal phenotype (Agholme et al., 2010). Cells were permeabilized for 10 min with 0.1% Triton X-100 (1.086.031.000, Merck Millipore) and subsequently left in a blocking buffer of 10% horse serum (HS; 16050-122, Thermo Fisher Scientific) in PBS for 15 min, incubated for 1 h with 1:200 primary antibody and 1% HS in PBS, incubated for 1 h with 1:200 secondary antibody in PBS and incubated for 30 min with 2 drops ml^–1^ of Actingreen (R37110, Thermo Fisher Scientific) and 2 drops ml^–1^ of NucBlue (R37606, Thermo Fisher Scientific) in PBS to stain for F-actin and cell nuclei, respectively. Samples were washed three times with PBS prior to each of the described steps of the staining. Images were obtained using the EVOS FL microscope (Thermo Fisher Scientific).

The cells in 3D hydrogel scaffolds were also immunofluorescently stained. Permeabilization was performed for 1 h using 0.1% Triton X-100 in PBS, followed by washing three times for 30 min with PBS. Then, a blocking buffer was applied for 4 h using 10% FBS in PBS, followed by overnight incubation of the primary antibody at 1:100 dilution in 10% FBS in PBS. Cells were washed four times for 2 h using 10% FBS in PBS and incubated overnight with 1:100 secondary antibody with 10% FBS in PBS. Actingreen and NucBlue were both added to PBS at 2 drops ml^–1^ that was used for the overnight incubation of the secondary antibody to stain for F-actin and cell nuclei, respectively. Afterward, samples were rinsed four times for 1 h with PBS. Z-stack images were obtained using a confocal laser scanning microscope (Leica TCS SP5X, Leica, Milton Keynes, United Kingdom).

### Quantification of Image-Based Parameters

We previously developed an image-based screening method for quantifiable assessment of neuronal cell cultures in 2D for comparing flat and nanogrooved substrates (Bastiaens et al., 2018b). In brief, the method uses the commercially available software package HCA-Vision (CSIRO, Australia) in combination with a Frangi vesselness algorithm to detect cell bodies, their respective neurites and the direction within the image of the neurites. Previously, we have shown the method can also be applied to 3D neuronal cell cultures (Bastiaens et al., 2019b).

Here, the method was applied to images collected from the 2D and 3D cultures being performed in the distinct culture microenvironments described in Section “Microbioreactor Fabrication”— “Microtunnel Device Fabrication” assessing the number of cells and the total neurite length. For the 3D microenvironments, the choice was made to select the slices from the *z*-stack that showed the cells on the substrate and into the 3D hydrogel microenvironment up to approximately 25 μm, with each slice thickness approximately 1.3 μm. To take into account that cell bodies and neurites may turn up in multiple slices of a *z*-stack, the mean number of cells and total neurite length was taken across the slices.

### Statistical Analysis

To be able to compare the samples of the different groups with different culture configurations, the values found for the number of cells and total neurite length were normalized to a surface area of 1 mm^–2^. The mean and standard deviation were calculated per group for the two normalized parameters. The normalization also allows for an analysis of variance. As the values within each group cannot be considered to follow a normal distribution, we have used the Kruskal–Wallis test with the *post hoc* Dunn’s test to assess whether the difference between the groups would be statistically significant. The analysis of variance was performed using Matlab R2018b (Mathworks).

## Results

### Fabrication

**FIGURE 2:**
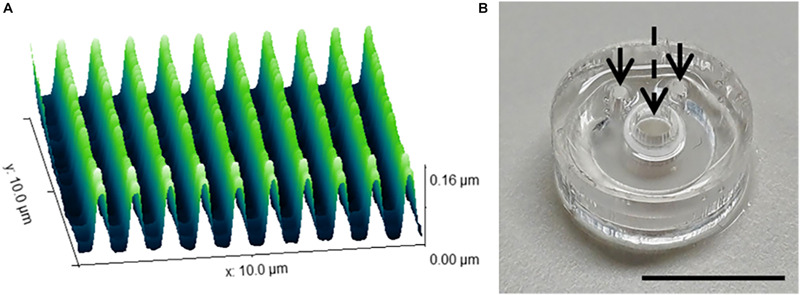
Nanogrooved pattern and microbioreactor fabrication for 2D and 3D neuronal cell culture devices. **(A)** Atomic force microscopy image of a 10 × 10 μm^2^ area of nanogrooves with a pattern period of 990 ± 22 nm and a ridge width of 246 ± 22 nm (*n* = 5) as used in the cell culture experiments with nanogrooved substrates. With permission from the open access article of Bastiaens et al. (2019b, Figure 3A) under the CC-BY license. **(B)** A microbioreactor polydimethylsiloxane (PDMS) gasket with cylindrical polyehtersulfone (PES) tube prior to bonding the microbioreactor to a glass cover slip. The arrows denote the inlets for the microfluidic channel, the dashed arrow denotes the cell culture chamber in the center of the PES tube for which a PES tube acts as a membrane to exchange nutrients and waste with the microfluidic channel. Scale bar denotes 1 cm. With permission from the open access article of Bastiaens et al. (2018a, Figure 2C) under the CC-BY license.

The range of 2D and 3D devices were fabricated using the methods described in Section “Materials and Methods.” The dimensions of the nanogrooved patterns used in the 2D PDMS substrates, 3D PDMS culture devices and the microtunnel devices were verified using AFM (Figure 2A). Results showed good fidelity, with patterns being approximately similar in size with regard to pattern period, ridge width and nanogroove height.

The microbioreactors (Figure 2B) served as the devices with a 3D microenvironment with flat substrates. Bonding of the microbioreactors to glass cover slips was successfully performed, with microbioreactors remaining bonded to the cover slips for the duration of the experiments.

**FIGURE 3:**
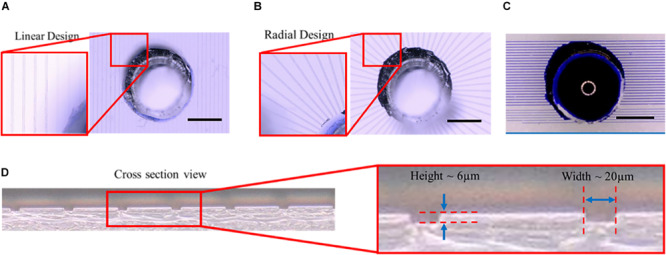
Microtunnel devices with linear and radial configuration. **(A)** Microtunnel device made of polydimethylsiloxane (PDMS) with microtunnels in a linear configuration, i.e., tunnels in parallel with inlets punched through the PDMS to access the tunnels. **(B)** Microtunnel device made of polydimethylsiloxane (PDMS) with microtunnels in a radial configuration, with a center inlet from which the tunnels extend outwards. **(C)** An example of a leakage test for the linear microtunnel configuration using a water-soluble dye. The dye was place on the inlet to check for any leakage points, showing proper bonding between the PDMS components of the device. Scale bars in **(A–C)** denote 1 mm. **(D)** A cross-section was cut of a microtunnel device to verify the dimensions of the microtunnels, showing a height of 6 μm and a width of 20 μm for the example shown.

The microtunnel devices with both the linear and radial configurations were made using photolithography and soft lithography. The resulting microtunnel structures in PDMS (Figures 3A,B) were clearly visible with brightfield microscopy, showing also that punching a hole for the inlets through the PDMS of the device properly connected the microtunnels to the inlets. A cross-section of the PDMS with microtunnels (Figure 3D) revealed that the tunnel height was measured to be 6 μm, which is very close to the intended height of 5 μm, whilst microtunnel widths were verified to be the same dimensions as designed for, i.e., 20 μm, 15 and 10 μm. Punching inlet and outlets results in relatively rough surfaces, which makes it not ideal for a cell culture microwell and the optical observation of the cells at the orifice of the microtunnel. Nevertheless, the geometry is sufficiently defined for a proof-of-principle. Also, to verify whether the microtunnels allowed for liquid flow without leakage, a linear (Figure 3C) microtunnel device was filled with dye. The dye flows through the microtunnels by capillary force and no leakage into other areas of the device was observed.

### SH-SY5Y Cell Culture

**FIGURE 4:**
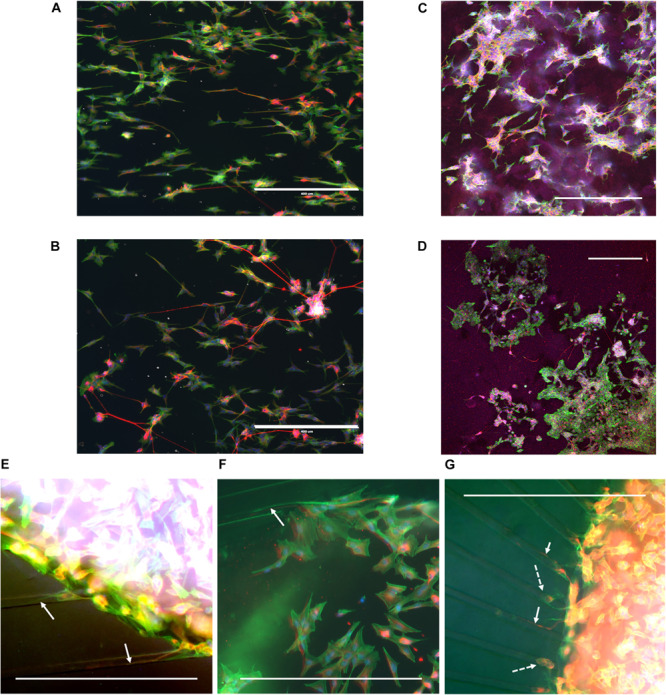
Example overview of SH-SY5Y cells in a range of different 2D and 3D microenvironments. SH-SY5Y cells were cultured for 21 days *in vitro* (DIV) in a series of different microenvironment containing either flat or nanogrooved substrates and consisted of either a 2D cell culture or a 3D cell culture in a hydrogel microenvironment on top of a substrate. Cells were differentiated into the neuronal phenotype on 0–3 DIV using retinoic acid and at 4 DIV with brain-derived neurotrophic factor. Cells were cultured on a nanogrooved substrate in 2D **(A)**, on a flat substrate in 2D **(B)**, on a nanogrooved substrate with a 3D hydrogel microenvironment **(C)**, on a flat substrate with a 3D hydrogel microenvironment **(D)**, in a microtunnel device with a nanogrooved substrate **(E)**, a microtunnel device with a flat substrate **(F)**, and a microtunnel device with a radial configuration and a flat substrate **(G)**. Immunofluorescence staining shows neuron-specific staining β-Tubulin III in red, F-actin in green and cell nuclei in blue. Scale bars denote 400 μm. White arrows highlight neurites that have grown into microtunnels, white dashed arrows show cell bodies present in microtunnels. Image contrast and brightness was increased for **(E–G)** to increase visibility of neurites within the images.

During cell culture, the SH-SY5Y cells were monitored for cell survival and differentiation. Overall, cells could be observed to survive for the duration of the experiment and extend neurites into their environment. Immunofluorescence staining revealed that cells had differentiated into the neuronal phenotype in all the realized microenvironments of the fabricated devices (Figure 4). The influence of the nanogrooved pattern on the direction of the alignment could be seen particularly well for the cells on the nanogrooved PDMS substrates for the 2D and 3D cell culture (Figures 4A,C), but was less pronounced for the other devices (Figures 4B,D–G). Although cells did differentiate and grew neurites in the microtunnel devices, only few neurites extended into the microtunnels themselves. These neurites had lengths in the range of 50–300 μm.

### Quantification of Image-Based Parameters

An overview was made of the images of the 2D and 3D devices for which image-based screening was performed to quantify the total number of cells and total neurite length present in the image (Table 1). These results were established based on previous data sets (referenced in Table 1), however, in the cases of the 2D and 3D cell cultures on flat substrates, the data shown here were not previously quantified or assessed in this context nor compared against the values shown for the 2D and 3D cell cultures on nanogrooved substrates. The results show that for a normalized surface area of 1 mm^2^ the mean number of cells is 275 cells. The Kruskal–Wallis test resulted in *p* = 0.9717, therefore no statistical significant differences were found between 2D or 3D cultures, or nanogrooved or flat substrate. For the 2D cell cultures, the total neurite length on 1 mm^2^ of a nanogrooved substrate is 6592 ± 2891 μm with higher values reaching up to 11926.1 μm, and for the flat substrate 3598.7 ± 1300.0 μm with lower values up until 1177.9 μm. The total neurite length for the 3D cell culture on 1 mm^2^ of nanogrooved substrate is 4490.2 ± 2686.0 μm, where one experiment showed 940.1 μm of total neurite length. The total neurite length for the 3D cell culture on 1 mm^2^ of flat substrate was 513.8 ± 257.8 μm with one experiment having a lower total neurite length of 149.2 μm. The nanogrooved substrates show a tendency toward higher totals for neurite length compared to the flat substrates, in particular for the comparison against the two groups of 3D cell culture. At *p* = 0.0281, the Kruskal–Wallis test showed statistically significant differences could be found, with the Dunn’s test revealing that neurites are longer in 2D cell cultures on nanogrooves as compared to 3D cell cultures on flat substrates.

**TABLE 1:** Parameter values derived from 2D and 3D SH-SY5Y cell cultures on nanogrooved and flat substrates.

Sample no.	Number of cells	Total neurite length [μm]	Est. number of cells in 1 mm^2 1^	Est. total neurite length [μm] in 1 mm^2 1^
**(I) 2D SH-SY5Y cell culture on nanogrooves (Bastiaens et al., 2019b)**				
Image surface area: 0.91 mm^2^				
1	227	4083	249	4487.0
2	138	4017	152	4414.5
3	289	4298	318	4723.0
4	299	10853	329	11926.1
5	264	6744	290	7411.3
Mean ± standard deviation:			268 ± 64	6592.4 ± 2891.0
**(II) 2D SH-SY5Y cell culture on flat substrate (Bastiaens et al., 2018b)**				
Image surface area: 0.91 mm^2^				
1	75	1072	82	1177.9
2	204	4384	224	4817.5
3	436	3057	479	3359.1
4	202	3877	222	4259.9
5	378	3985	415	4379.1
Mean ± standard deviation:			284 ± 144	3599.7 ± 1300.0
**(III) 3D SH-SY5Y cell culture on nanogrooves (Bastiaens et al., 2019b)**				
Image surface area: 0.60 mm^2^				
1	197^2^	4636.5^2^	216	5095.1
2	352^2^	6766.2^2^	386	7435.4
3	159^2^	855.5^2^	175	940.1
Mean ± standard deviation:			259 ± 91	4490.2 ± 2686.0
**(IV) 3D SH-SY5Y cell culture on flat substrates (Bastiaens et al., 2018a)**				
Image surface area: 2.40 mm^2^				
1	751^2^	1681^2^	313	699.6
2	278^2^	358.5^2^	116	149.2
3	1004^2^	1664^2^	418	692.6
Mean ± standard deviation:			282 ± 125	513.8 ± 257.8

*^1^For comparison between images from each category, the values for the number of cells and total outgrowth length were divided by the image surface area corresponding to the appropriate category. ^2^The mean values were taken for the number of cells and the total outgrowth length for analyzed slices of each z-stack sample for a height of approximately 25 μm starting from the substrate surface.*

## Discussion

Advanced *in vitro* models are a necessity to better understand brain physiology and pathology. With the vast array of available micro- and nanofabrication methods, new BOC technology can be designed that provides the means to maintain, manipulate and analyze neuronal cells beyond what is capable with conventional cell culture methods. Here, we have investigated how nanogrooved patterns can be implemented as part of 2D and 3D neuronal cell culture and in designing such systems whether we can find baseline expectations for parameters such as the total neurite length that aid in design optimization. Also, we have developed microtunnels devices that are bonded to nanogrooved patterns and provided a proof of principle that it is feasible to benefit from the nanogroove alignment in such a device.

Previously generated datasets were employed to gain new insights into what the data indicates about the neuronal differentiation in the 2D and 3D microenvironments we have developed. Specifically, we compared different experiments within a range of reasonable similarities with regard to the chosen cell line, cell seeding densities and analysis method. The quantified results for the total number of cells and total neurite length per normalized area of 1 mm^2^ show that similar ranges of values were found across the experiments with the notable exception of the total neurite length as found in the 2D cell culture on nanogrooves substrates versus 3D cell cultures on flat substrates. Considering the limited available data at this time, the statistical assessment can be used as an indication for future experiments which employ similar culturing protocols. Considering the evidence in literature and our previous work for the influence of nanotopography on neuronal cells, in particular neurite length and direction (Johansson et al., 2006; Bremus-Koebberling et al., 2012), the data here confirm nanogrooves can direct and extend the length of neurites.

The comparison made in Table 1 helps us in formulating a baseline against which future experiments can be compared and such a repository of data can also be advantageous toward software approaches that give rise to more insight in experimental results. For instance, machine learning and big data approaches using artificial intelligence can use such data sets to improve prediction quality, effectively establishing parameters that reveal a ‘ground truth,’ which has for instance been shown to aid in identification of unknown metabolites in human cohorts (Quell et al., 2017). Whilst the mentioned study is based on a different topic and performed at a different scale, such machine learning approaches using established parameters may lead to faster and more robust BOC design optimization.

The microtunnel devices were fabricated and tested for technical feasibility. Whilst culture of SH-SY5Y cells was feasible in the system and the cells showed presence of β-Tubulin III and neurites, the cells and their neurites remained mostly within the inlets in which they were seeded in the microfluidic protocol used here. However, evidence of outgrowth into the 3D structure of the microtunnels was preliminary demonstrated. The design of the system was limited by the manner of creating the inlets, as the biopsy punch used to create the inlets resulted in rough edges that made visualization of the cells seeded near the microtunnels challenging. Several improvements can be considered for the next generation of device, like punching the inlets and outlets from the side of the microtunnels starting with the sharp edge of the biopsy tool at the critical point of observation. Alternatively, a two-level SU-8 master structure can be considered. Also, the manual handling required to punch the inlets in the microtunnel devices meant that the distance between inlets was several mm in distance. Due to the volume size of the inlets, being 2 mm in diameter and approximately 5 mm high, there was a risk of these microliter volumes to evaporate if sufficient medium was not provided to the cells. Therefore, we chose to submerge the microtunnels devices in medium, which held us from utilizing either hydrostatic pressure difference or chemical gradients. Together, these choices most likely limited the cells to extend their neurites into the microtunnels instead of to their neighboring cells within that same inlet. Nevertheless, we expect that with improvements to the design that allow for closer interaction between inlets and the application of fluidic or chemical gradient will improve our findings. Future experiments will need to demonstrate whether a nanogrooved substrate added underneath the microtunnel device indeed aids in extended growth of neurites through the microtunnels. Furthermore, experiments with the radial microtunnel devices may be more suited to facilitate organoid cultures compared to the linear configuration. A radial microtunnel design will allow for organoids, which tend to be somewhat spherical in shape, to connect to other compartments at the circumference of the central compartment in all directions evenly. Incorporating also nanogrooves in a radial configuration of the microtunnel device would be an interesting future direction of such experiments since the angle of alignment of nanogrooves with the microtunnels would systematically vary, which may offer also novel assay capabilities of such a Brain-on-a-Chip device. Here too, measuring total neurite length across the microtunnels can help to optimize this type of BOC based culture protocol.

In perspective of a biological study design, the indicator for neurite length is suitable to a range of nanogroove-related experiments using SH-SY5Y cells and the novel combination of nanogrooves with microtunnel devices, and when gathered as shown here, this advances the BOC toolbox. For example, we strive to design a BOC platform that allows for analysis of multiple neuronal regions, here the midbrain, peripheral and enteric neurons, and link these to each other via clearly compartmentalized regions. Measuring total neurite length then can help to optimize the device and culture protocols to ensure control and reproducibility of a BOC system prior to setting up a biological study in replicas. With this in mind, we do expect that extending the indicative results for neuronal cell cultures in 2D and 3D on nanogrooved and flat substrates should also be extended toward human induced pluripotent stem cell-derived neurons. We have previously performed experiments with such cells on nanogrooves (Bastiaens et al., 2019a), however, this was done in the context of their electrophysiological assessment using calcium imaging, not on morphological features such as neurite length. With the addition of clear expectations for valuable parameters that indicate neuronal differentiation and the potential to implement human induced pluripotent stem cells for more realistic cultures, such a platform lends itself to preclinical drug discovery where both healthy and patient-derived cells can be cultured and analyzed within the BOC platform. Another potential benefit of this type of microfluidic platform, is the use of the radial configuration, where it would be feasible to insert brain organoids and microfluidically connect these to other compartments within a BOC platform.

To summarize, we show here an overview of different methods to create 2D and 3D microenvironments which can influence neuronal differentiation. However, we still need to quantify in more detail the outgrowth phenomena of neuronal cell networks in our new microenvironments supplied by a combination of micro- and nanofabrication as described in this paper. In more detail, the current exploration of culture protocols in the new devices using SH-SY5Y cells allows to assess total neurite length with a baseline value of approximately 200–400 cells and 4,000–5,000 μm neurite length per 1 mm^2^ at 21 DIV. We also show that our microtunnel devices can be used for neuronal cell cultures, but do not yet result in observable differences in neurite growth between microtunnels with or without nanogrooved substrates. These initial observations still help us to find merits in baseline measurements of total outgrowth (here: SH-SY5Y cells) to determine the quality and the effect that therapeutic agents or toxins have on these neuronal cell cultures. Also, these experiments show the importance of a diverse BOC toolbox with varying approaches in micro- and nanotechnology to enhance *in vitro* brain models.

## Data Availability Statement

All datasets generated for this study are included in the article/supplementary material.

## Author Contributions

RL acquired the funding. AB, RS-K, and RL conceptualized and designed the manuscript, interpreted the data, prepared the manuscript, and contributed to the final approved version of the manuscript. AB and RS-K acquired and analyzed the data. All authors contributed to the article and approved the submitted version.

## Conflict of Interest

The authors declare that the research was conducted in the absence of any commercial or financial relationships that could be construed as a potential conflict of interest.

## Abbreviations

2D: two-dimensional
3D: three-dimensional
BDNF: brain-derived neurotrophic factor
BOC: brain-on-chip
DIV: days *in vitro*
DMEM: Dulbecco’s modified Eagle’s medium
FBS: fetal bovine serum
OOC: organ-on-chip
PBS: phosphate buffered saline
PDMS: polydimethylsiloxane
PES: polyethersulfone
PMMA: poly(methyl methacrylate)
RA: retinoic acid.
